# The Effect of Lymph Node Harvest on Prognosis in Locally Advanced Middle-Low Rectal Cancer After Neoadjuvant Chemoradiotherapy

**DOI:** 10.3389/fonc.2022.816485

**Published:** 2022-02-15

**Authors:** Zhuangbin Lin, Xiaobo Li, Jianyuan Song, Rong Zheng, Cheng Chen, Anchuan Li, Benhua Xu

**Affiliations:** ^1^ Department of Radiation Oncology, Fujian Medical University Union Hospital, Fuzhou, China; ^2^ The Graduate School, Fujian Medical University, Fuzhou, China; ^3^ Department of Radiation Oncology, Fujian Branch of Shanghai Children’s Medical Center Affiliated to Shanghai Jiaotong University School of Medicine, Fuzhou, China; ^4^ Department of Radiation Oncology, Fujian Children’s Hospital, Fuzhou, China; ^5^ Department of Medical Imaging Technology, College of Medical Technology and Engineering, Fujian Medical University, Fuzhou, China; ^6^ Union Clinical Medicine College, Fujian Medical University, Fuzhou, China; ^7^ School of Clinical Medicine, Fujian Medical University, Fuzhou, China

**Keywords:** locally advanced rectal cancer, neoadjuvant chemoradiotherapy, lymph nodes retrieved, prognosis, PLN, LNR

## Abstract

**Objective:**

The purpose of this study was to investigate the relationship between lymph node harvest and the prognosis in locally advanced rectal cancer (LARC) patients after neoadjuvant chemoradiotherapy (nCRT).

**Methods:**

Patients who were diagnosed with clinical LARC and treated with nCRT and radical surgery between June 2008 and July 2017 were included in this study. The relationship between lymph node retrieval and prognosis was analyzed. Other lymph node-related indicators were explored.

**Results:**

A total of 837 patients with a median follow-up of 61 (7-139) months were included in the study. The five-year DFS and OS rates of all patients were 74.9% and 82.3%, respectively. Multivariate survival analysis suggested that dissection of ≥ 12 lymph nodes did not improve OS or DFS. 7 was selected as the best cutoff value for the total number of lymph nodes retrieved by Cox multivariate analysis (χ2 = 10.072, HR: 0.503, P=0.002). Dissection of ≥ 5 positive lymph nodes (PLNs) was an independent prognostic factor for poorer DFS (HR: 2.104, P=0.004) and OS (HR: 3.471, p<0.001). A positive lymph node ratio (LNR) of more than 0.29 was also an independent prognostic factor for poorer DFS (HR: 1.951, P=0.002) and OS (HR: 2.434, p<0.001).

**Conclusion:**

The recommends that at least 7 harvested lymph nodes may be more appropriate for LARC patients with nCRT. PLN and LNR may be prognostic factors for LARC patients with ypN+ after nCRT.

## Introduction

Globally, the incidence of colorectal cancer ranks third among all malignant tumors, accounting for 8.0% to 9.0% of all new cases, and it is the third leading cause of cancer deaths worldwide (1). Neoadjuvant chemoradiotherapy (nCRT) followed by total mesorectal excision (TME) is recommended as a standard treatment mode for locally advanced rectal cancer (LARC) in the National Comprehensive Cancer Network’s (NCCN) Clinical Practice Guidelines (2). Regional lymph node status is an important factor affecting overall survival (OS) and disease-free survival (DFS) in patients with nonmetastatic rectal cancer (RC) (3).

In the current TNM staging system, rectal cancer with regional lymph node metastasis is classified into stage III (4). According to the current guidelines of the Union for International Cancer Control (UICC) and the American Joint Committee on Cancer (AJCC), at least 12 lymph nodes retrieved during surgery are required to determine the presence or absence of lymph node metastasis and improve the accuracy of postoperative staging of rectal cancer (4–6). However, these conclusions are derived from patients with rectal cancer who have not received neoadjuvant treatment and have undergone radical surgical resection as the initial treatment. Many previous studies have proposed that the harvest rate of lymph nodes in rectal cancer patients receiving neoadjuvant therapy is significantly lower than that in patients undergoing surgical resection as the initial treatment (7–9). The study of Baxter et al. (10) showed that less than 20% of patients with stage II rectal cancer who received preoperative radiotherapy had more than 12 lymph nodes detected during the operation. For these patients, it is unclear whether the reduction in the number of lymph nodes detected will lead to insufficient staging or affect the prognosis. At present, the medical community has not reached a consensus on the requirements for the total number of lymph nodes retrieved from patients undergoing neoadjuvant therapy.

In this study, we retrospectively analyzed the clinical data of 837 LARC patients who underwent nCRT in our center from June 2008 to July 2017 and explored the relationship between lymph node detection and prognosis after nCRT combined with TME.

## Materials and Methods

### Study Population

The data of 1083 LARC patients who underwent neoadjuvant chemoradiotherapy in our center from June 2008 to July 2017 were collected according to the following inclusion criteria (see below). A total of 246 patients were excluded from the study based on the exclusion criteria (see below). A total of 837 patients were finally included. All cases had records of imaging data: 808 patients underwent MRI, 811 patients underwent endoscopic US and 790 patients underwent both. All cases were followed up regularly through outpatient clinics or by telephone until 2020.04.23.

The inclusion criteria were as follows: 1. Patients who were pathologically diagnosed with rectal cancer by colonoscopy and received treatment for the first time; 2. Complete medical history data; 3. According to the 7th edition of the AJCC/UICC TNM staging system (patients diagnosed before 2010 were restaged according to the 7th edition staging system), patients were clearly diagnosed with clinical stage II/III LARC after completing the preoperative imaging examinations; 4. According to preoperative MR, colonoscopy and digital rectal examination, the lower edge of the tumor was ≤12 cm from the anal edge; 5. Patients who received neoadjuvant therapy and laparoscopic radical surgery for rectal cancer.

The exclusion criteria were as follows: 1. Patients with a history of other organ tumors or those diagnosed with simultaneous/metachronous multisource cancer; 2. Patients who accepted neoadjuvant radiotherapy/chemotherapy alone or received sequential chemotherapy; 3. Distant metastasis was detected before or during the operation; 4. A previous history of pelvic radiotherapy or surgery; 5. Patients received open surgery.

### Treatment

#### Neoadjuvant Chemoradiotherapy

Radiotherapy was administered with 3-dimensional conformal radiation therapy (3D-CRT), intensity modulated radiation therapy (IMRT) or volumetric modulated arc therapy (VMAT) techniques. Radiotherapy treatments were planned using the Varian Eclipse treatment planning system, version 13.5. The delineation of the target and the prescription dose followed the 62 and 83 International Commission on Radiation Units and Measurements (ICRU) reports. The definition of the target regions was as follows: gross tumor volume (GTV) was defined as the volume of the primary tumor (GTV-T) and metastatic lymph nodes (GTV-N) determined by CT imaging before treatment. The clinical target volume (CTV) was defined as GTV plus all mesorectum, presacral soft tissue, internal iliac and obturator lymphatic drainage regions. The planning target volume (PTV) was defined as the GTV or CTV with uniform margins of 5 mm. All patients received a long-term radiotherapy regimen: a total dose of 50 Gy/25 F (2.0 Gy/F) was delivered to PTV-GTV, and a total dose of 45 Gy/25 F (1.8 Gy/F) was delivered to PTV-CTV. The doses to the normal tissues were limited according to the RTOG pelvic normal tissue contouring guidelines. Irradiation was performed continuously for 5 days a week with weekends off. The total duration of treatment was 5 weeks.

All patients received 5-fluorouracil (5-FU)-based concurrent chemotherapy during neoadjuvant radiotherapy. There were two main concurrent chemotherapy regimens: 1. regimens containing oxaliplatin, including the XELOX, FOLFOX4, mFOLFOX6 and FOLFOX6 regimens; and 2. regimens not containing oxaliplatin, including a single agent capecitabine regimen and a De Gramont regimen.

#### Surgery and Pathology

Radical resection surgery was performed approximately 8–12 weeks after completion of nCRT. All operations were conducted following the principles of TME. The surgeon decides the surgical procedure, surgical extent and anus preservation according to the size, location and extent of the tumor, as shown in the relevant preoperative imaging examinations. The criteria of lymph node collection and counting were as follows: 1. To meet the requirements of lymph node dissection in TME surgery, at least 3 sites of lymph nodes, including pararectal lymph nodes, the lymph nodes adjacent to the superior rectal artery and the lymph nodes around the root of the inferior mesenteric artery, were removed during the operation; 2. The tissue samples obtained during the operation were manually dissected by the surgeons to find and collect the lymph nodes; 3. Samples were immersed in 10% formalin after resection and sent to the pathology department on the same day. Pathological examinations and diagnoses of the samples were performed independently by 2 experienced pathologists. In addition to the lymph nodes removed by the surgeons, the pathologists tried to collect residual lymph nodes from the other samples. Pathological TNM stage classification was evaluated based on the 7th edition of the UICC/AJCC TNM staging system. To assess the response to treatments, tumor regression grade (TRG) is used to evaluate histologic tumor regression after CRT. AJCC TRG classification system (grade 0, no sign of tumor cells; grade 1, single cell or small groups of cancer cells; grade 2, residual cancer with a desmoplastic response; grade 3, no regression) was used in this study (11). Positive lymph node (PLN) was defined as the number of positive lymph nodes after surgery. The positive lymph node ratio (LNR) was calculated as the quotient between the number of tumor-infiltrated lymph nodes and total number of retrieved lymph nodes.

### Follow-Up

Patients were reviewed once every 3 months within the first 2 years after surgery, then every 6 months for the next 3 years and annually thereafter. The re-examinations included a physical examination (particularly a digital rectal examination), serum carbohydrate antigen 199 (CA199), carcinoembryonic antigen (CEA), chest CT scanning, liver plus pelvic MRI, transrectal color Doppler ultrasound, colonoscopy and other necessary examinations. The last follow-up date was April 23, 2020. Disease-free survival (DFS) was defined as the time from the date of surgery to the date of disease recurrence (locoregional or distant recurrence) or death from any cause. Overall survival (OS) was defined as the time from the date of surgery to the date of death from any cause.

### Statistical Analysis

Statistical analysis was performed using SPSS (IBM SPSS Statistics for Windows, Version 23.0) and Stata (StataCorp 2013, Stata Statistical Software for Windows, Release 13). X-tile (Yale University, X-tile Bioinformatics Software for Windows, Version 3.6.1) was used to select the best cutoff value for survival analysis. For comparisons between groups, the chi-square test was used for categorical variables, while the Mann–Whitney rank sum test or Kruskal–Wallis rank sum test was used for nonnormally distributed continuous variables. Survival curves were drawn by the Kaplan–Meier method and compared using log-rank tests. The independent prognostic factors for rectal cancer were identified by the Cox proportional hazard regression model. Before Cox analysis, it was necessary to perform collinearity diagnosis and test proportional hazards (PH) assumptions for the included analysis factors. For the variables with violations of the PH assumptions, time-dependent Cox regression was applied. The independent variable was directly included in the Cox analysis if it did not meet the time-dependent characteristics; otherwise, it was used as a stratifying variable for stratified Cox analysis. The most significant statistical cutoff point for the total number of lymph nodes retrieved was determined by a cutoff point survival analysis. The likelihood ratio chi-square was calculated with Cox regression to measure homogeneity, and a higher likelihood ratio chi-square score indicates better homogeneity. P < 0.05 was considered statistically significant unless specified otherwise.

## Results

A total of 837 patients with stage II/III rectal cancer who underwent nCRT and TME in our center from June 2008 to July 2017 were included in this study. The median follow-up was 61 (7~139) months. The clinical and pathological data of the patients are shown in **Table 1**. A total of 93.9% of patients were clinical stage III before treatment. The median number of lymph nodes detected was 12 (0~42). A total of 373 (44.6%) patients had fewer than 12 lymph nodes harvested, and 464 (55.4%) patients had 12 or more lymph nodes retrieved.

**Table 1:** Participant characteristics.

Demographic and clinicopathologic features	n, %	<12 lymph nodes retrieved (n, %)	≥12 lymph nodes retrieved (n, %)	*P*
**Gender**				0.312
Male	550 (65.7)	252 (67.6)	298 (64.2)	
Female	287 (34.3)	121 (32.4)	166 (35.8)	
**Age**				0.005*
≤65	675 (80.6)	285 (76.4)	390 (84.1)	
>65	162 (19.4)	88 (23.6)	74 (15.9)	
**Clinical stage**				0.016*
II	51 (6.1)	31 (8.3)	20 (4.3)	
III	786 (93.9)	342 (91.7)	444 (95.7)	
**Pretreatment CEA**				0.974
≤5	455 (54.4)	203 (54.4)	252 (54.3)	
>5	382 (45.6)	170 (45.6)	212 (45.7)	
**Pretreatment CA199 (ng/ml)**				0.784
≤37	730 (87.2)	324 (86.9)	406 (87.5)	
>37	107 (12.8)	49 (13.1)	58 (12.5)	
**Location**				0.185
Low	637 (76.1)	292 (78.3)	345 (74.4)	
Middle	200 (23.9)	81 (21.7)	119 (25.5)	
**nCRT regimens**				0.002*
Non oxaliplatin-containing	541 (64.6)	220 (59.0)	321 (69.2)	
Oxaliplatin-containing	296 (35.4)	153 (41.0)	143 (30.8)	
**Consolidation chemotherapy**				0.115
No	569 (68.0)	243 (65.1)	326 (70.3)	
Yes	268 (32.0)	130 (34.9)	138 (29.7)	
**Adjuvant chemotherapy**				0.105
No	174 (20.8)	87 (23.3)	87 (18.8)	
Yes	663 (79.2)	286 (76.7)	377 (81.3)	
**Radiation therapy**				0.215
3D-CRT	254 (30.3)	111 (29.8)	143 (30.8)	
IMRT	555 (66.3)	245 (65.7)	310 (66.8)	
VMAT	28 (3.3)	17 (4.6)	11 (2.4)	
**Presurgery CEA**				0.715
≤5	723 (86.4)	324 (86.9)	399 (86.0)	
>5	114 (13.6)	49 (13.1)	65 (14.0)	
**Presurgery CA199**				
≤37	804 (96.1)	357 (95.7)	447 (96.3)	0.644
>37	33 (3.9)	16 (4.3)	17 (3.7)	
**Pathological stage**				0.001*
ypCR	170 (20.3)	85 (22.8)	85 (18.3)	
ypI	220 (26.3)	111 (29.8)	109 (23.5)	
ypII	233 (27.8)	105 (28.2)	128 (27.6)	
ypIII	214 (25.6)	72 (19.3)	142 (30.6)	
**ypT**				0.112
T0	177 (21.1)	88 (23.6)	89 (19.2)	
T1	51 (6.1)	25 (6.7)	26 (5.6)	
T2	222 (26.5)	106 (28.4)	116 (25.0)	
T3	348 (41.6)	136 (36.5)	212 (45.7)	
T4	39 (4.7)	18 (4.8)	21 (4.5)	
**ypN**				<0.001*
N0	623 (74.4)	301 (80.7)	322 (69.4)	
N1	175 (20.9)	65 (17.4)	110 (23.7)	
N2	39 (4.7)	7 (1.9)	32 (6.9)	
**Surgery**				0.061
Non anal preservation	87 (10.4)	47 (12.6)	40 (8.6)	
Anal preservation	750 (89.6)	326 (87.4)	424 (91.4)	
**AJCC TRG**				0.212
0	177 (21.1)	89 (23.9)	88 (19.0)	
1	280 (33.5)	126 (33.8)	154 (33.2)	
2	302 (36.1)	129 (34.6)	173 (37.3)	
3	78 (9.3)	29 (7.8)	49 (10.6)	

Asterisks indicate significance (*P < 0.05).

### The Response to nCRT and Its Involvement With the Total Number of Lymph Nodes Retrieved

**Figure 1:**
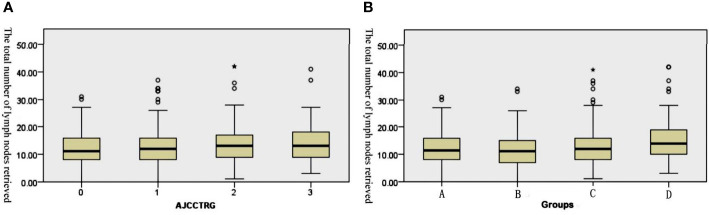
Box plots of the number of retrieved lymph nodes. **(A)** The retrieved lymph nodes in the four stage groups after the operation. **(B)** The retrieved lymph nodes in the different TRG groups after the operation.

Median total number of lymph nodes retrieved in the four TRG0-3 groups was 11 (0~31), 12 (0~37), 13 (1~42), and 13 (3~41) (P=0.011, **Figure 1A**). A cohort of patients with TRG0-1 was considered a good treatment response group, and another with TRG2-3 was considered a poor treatment response group. The total number of lymph nodes retrieved in the good response group was significantly lower than that in the poor response group, and the median total numbers were 12 (0~37) and 13 (1~42), respectively (P=0.001).

Based on the postoperative staging, all patients were divided into four groups: Group A (ypCR), Group B (ypT1-2N0), Group C (ypT3-4N0) and Group D (ypT1-4N+). The median total number of lymph nodes retrieved in the four groups was 11.5 (0~31), 11 (0~34), 12 (1~41) and 14 (3~42). The results of the Kruskal–Wallis tests revealed that there were significant differences in the total number of lymph nodes among the four subgroups (H=27.158, p<0.001). After pairwise comparison, the total number of lymph nodes retrieved in Group D was significantly higher than that in the other three groups (the adjusted P values were p<0.001, p<0.001 and P=0.017, respectively, **Figure 1B**). Further analysis showed that 612 (73.1%) patients achieved tumor downstaging after nCRT, while 225 (26.9%) patients did not achieve tumor downstaging. The median total number of lymph nodes retrieved in the two groups was 12 (0~41) and 13 (1~42), and the difference between them was statistically significant (U=55239.0, p<0.001).

### Survival Analysis

The 5-year DFS was 74.9%, and the 5-year OS was 82.3% for all patients in our cohort. Kaplan–Meier survival analysis showed that dissecting fewer than 12 lymph nodes was not significantly related to a poor DFS and OS (P=0.909 and 0.955, respectively). The results are detailed in **Table 2**.

**Table 2:** Univariate analysis of prognostic factors (OS and DFS) in 837 patients.

Demographic and clinicopathologic features	DFS			OS		
	5y %	standard errors	P	5y %	standard errors	P
**Gender**			0.473			0.255
Male	74.0	0.020		80.8	0.019	
Female	76.6	0.026		85.1	0.023	
**Age**			0.532			0.196
≤65	75.8	0.017		82.9	0.016	
>65	70.7	0.040		79.5	0.037	
**Clinical stage**			0.906			0.384
II	76.2	0.060		87.4	0.048	
III	74.8	0.016		81.9	0.015	
**Pretreatment CEA**			<0.001*			0.008*
≤5	80.4	0.019		86.1	0.018	
>5	68.5	0.025		77.8	0.024	
**Pretreatment CA199 (ng/ml)**			0.005*			0.001*
≤37	76.6	0.016		83.9	0.015	
>37	63.7	0.049		71.0	0.048	
**Location**			0.197			0.457
Low	73.7	0.018		81.2	0.017	
Middle	78.9	0.030		86.0	0.026	
**nCRT regimens**			0.092			0.074
Non oxaliplatin-containing	77.3	0.019		83.4	0.019	
Oxaliplatin-containing	71.0	0.027		79.7	0.024	
**Consolidation chemotherapy**			0.101			0.175
No	76.4	0.019		83.0	0.018	
Yes	71.9	0.028		80.7	0.026	
**Adjuvant chemotherapy**			0.048*			0.389
No	82.1	0.029		87.3	0.026	
Yes	73.3	0.018		81.4	0.016	
**Radiation therapy**			0.324			0.222
3D-CRT	71.8	0.028		79.6	0.026	
IMRT	76.4	0.019		82.8	0.019	
VMAT	78.6	0.078		89.3	0.058	
**Presurgery CEA**			0.007*			0.036*
≤5	76.3	0.016		83.3	0.015	
>5	65.8	0.047		75.4	0.047	
**Presurgery CA199**			0.137			0.090
≤37	75.3	0.016		82.6	0.015	
>37	65.3	0.086		74.3	0.079	
**Total number of lymph nodes retrieved**			0.909			0.955
<12	75.4	0.023		82.8	0.021	
≥12	74.5	0.021		81.7	0.020	
**Pathological stage**			<0.001*			<0.001*
ypCR	90.4	0.023		94.5	0.018	
ypI	87.0	0.024		92.2	0.020	
ypII	70.1	0.032		78.4	0.030	
ypIII	55.3	0.035		66.8	0.036	
**ypT**			<0.001*			<0.001*
T0	88.5	0.024		92.9	0.020	
T1	91.4	0.042		95.3	0.033	
T2	80.5	0.028		89.2	0.023	
T3	63.0	0.027		71.7	0.027	
T4	65.8	0.078		73.7	0.072	
**ypN**			<0.001*			<0.001*
N0	81.7	0.016		87.6	0.015	
N1	57.6	0.038		69.5	0.039	
N2	45.1	0.081		55.2	0.087	
**Surgery**			0.057			0.010*
Non anal preservation	72.2	0.048		75.3	0.051	
Anal preservation	75.3	0.016		83.1	0.015	
**AJCC TRG**			<0.001*			<0.001*
0	89.7	0.023		94.1	0.018	
1	75.0	0.028		81.7	0.026	
2	68.8	0.028		77.1	0.027	
3	64.5	0.055		75.9	0.050	

Asterisks indicate significance (*P < 0.05).

The stepwise multivariate Cox analysis for DFS showed that dissection of ≥ 12 lymph nodes was not an independent prognostic factor for DFS in the ≤ 65 age group (P=0.413 in the ≤ 65 age group and P=0.390 in the other group). For OS, the results remained nonsignificant in all patients (P=0.154). The results are detailed in **Table 3**.

**Table 3:** Stepwise multivariate Cox analysis for DFS and OS.

*The result for DFS (age ≤ 65)*				
Variable	HR	P value	Lower CI	Upper CI
**ypT**				
0	Ref	.	.	.
1	0.584	0.394	0.170	2.009
2	1.497	0.192	0.817	2.745
3	2.581	0.001*	1.472	4.524
4	2.367	0.026*	1.106	5.062
**ypN**				
0	Ref	.	.	.
1	2.609	<0.001*	1.843	3.692
2	3.329	<0.001*	1.981	5.595
***The result for DFS (age>65)***				
**Variable**	**HR**	**P value**	**Lower CI**	**Upper CI**
**Pretreatment CEA**	2.064	0.023	1.106	3.851
***The result for OS***				
**Variable**	**HR**	**P value**	**Lower CI**	**Upper CI**
**ypT**				
0	Ref	.	.	.
1	0.498	0.362	0.111	2.230
2	1.387	0.361	0.688	2.797
3	3.153	<0.001*	1.686	5.897
4	3.002	0.007*	1.358	6.636
**ypN**				
0	Ref	.	.	.
1	2.024	<0.001*	1.405	2.916
2	3.721	<0.001*	2.239	6.183
**Pretreatment CA199**	1.505	0.047	1.006	2.251

Asterisks indicate significance (*P < 0.05).

Subgroup survival analyses were conducted in the TRG0~1 and TRG2~3 groups separately. Univariate and multivariate survival analyses showed that dissection of ≥ 12 lymph nodes was not significantly associated with DFS (P=0.839 and P=0.330, respectively) or OS (P=0.601 and P=0.199, respectively) in the TRG0~1 group or in the TRG2~3 group (P=0.945 and P=0.552 for DFS, respectively; P=0.991 and P=0.641 for OS, respectively).

### New Lymph Node Related Indicators

#### The Best Cutoff Value of the Total Number of Lymph Nodes Retrieved

**Figure 2:**
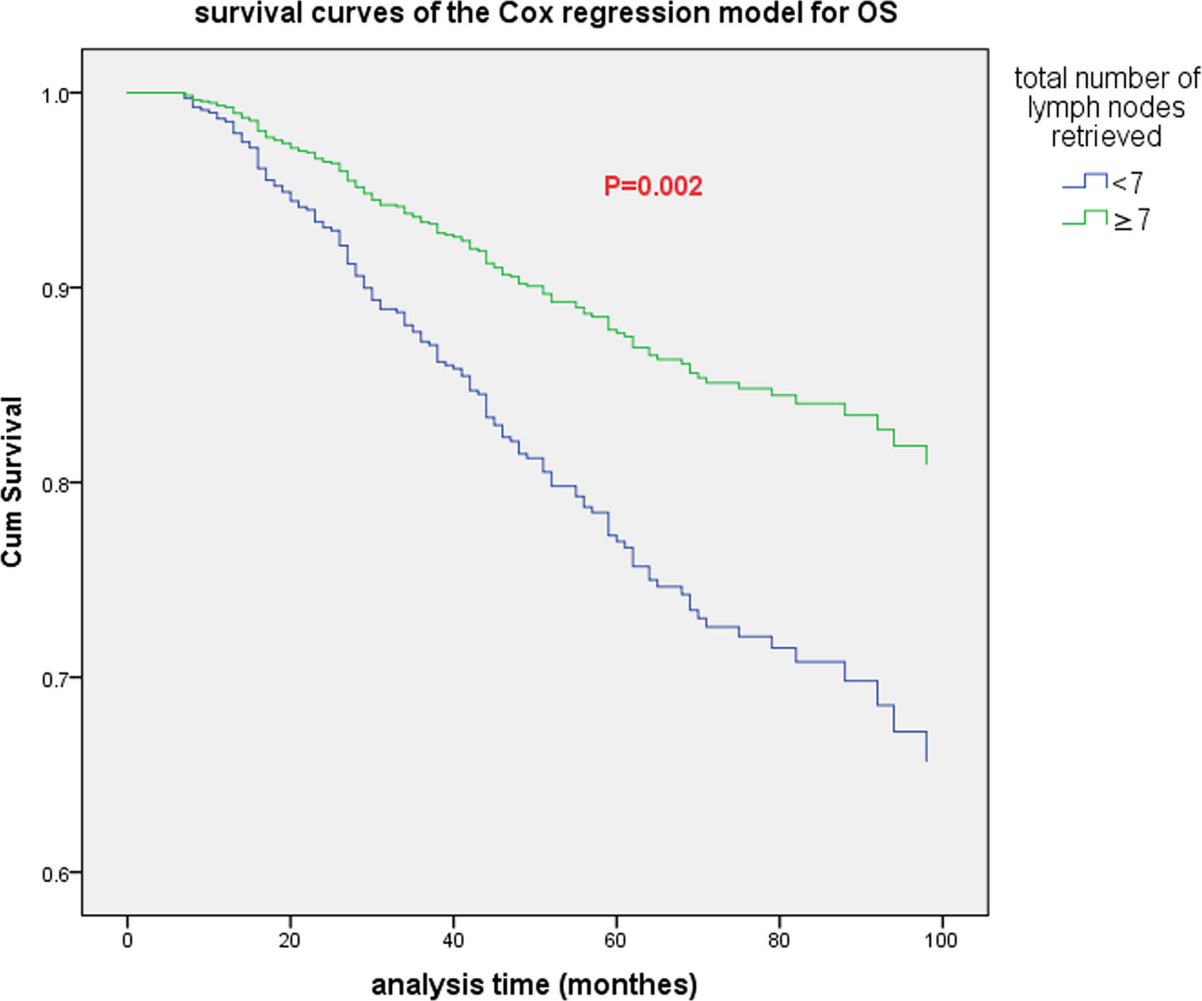
The survival curve (based on multivariate Cox regression) of all patients when the optimal cutoff value of the total number of lymph nodes retrieved was 7.

Cox multivariate analysis was adopted, and 1 to 11 were used as the cutoff values of the total number of lymph nodes retrieved in the model. The results indicated that there were significant differences in OS when the cutoff values were 3, 4, 6, 7, 9 and 11 (see **Table 4**). The highest chi-square value was deemed the cutoff point. Therefore, 7 was selected as the best cutoff value for the total number of lymph nodes retrieved. Survival curves based on multivariate Cox regression are shown in **Figure 2**.

**Table 4:** Cut-point survival analysis of each dissected lymph node.

Cutoff value	HR	χ2	P	Lower CI	Upper CI
1	–	–	0.410	–	–
2	–	–	0.863	–	–
3	0.314	4.926	0.026*	0.113	0.873
4	0.295	9.273	0.002*	0.135	0.647
5	–		0.079	–	–
6	0.563	4.467	0.035*	0.330	0.959
7	0.503	10.072	0.002*	0.329	0.769
8	–		0.109	–	–
9	0.633	5.910	0.015*	0.437	0.915
10	–		0.072	–	–
11	0.667	5.585	0.018*	0.477	0.933

Asterisks indicate significance (*P < 0.05).

#### Positive Lymph Node

**Figure 3:**
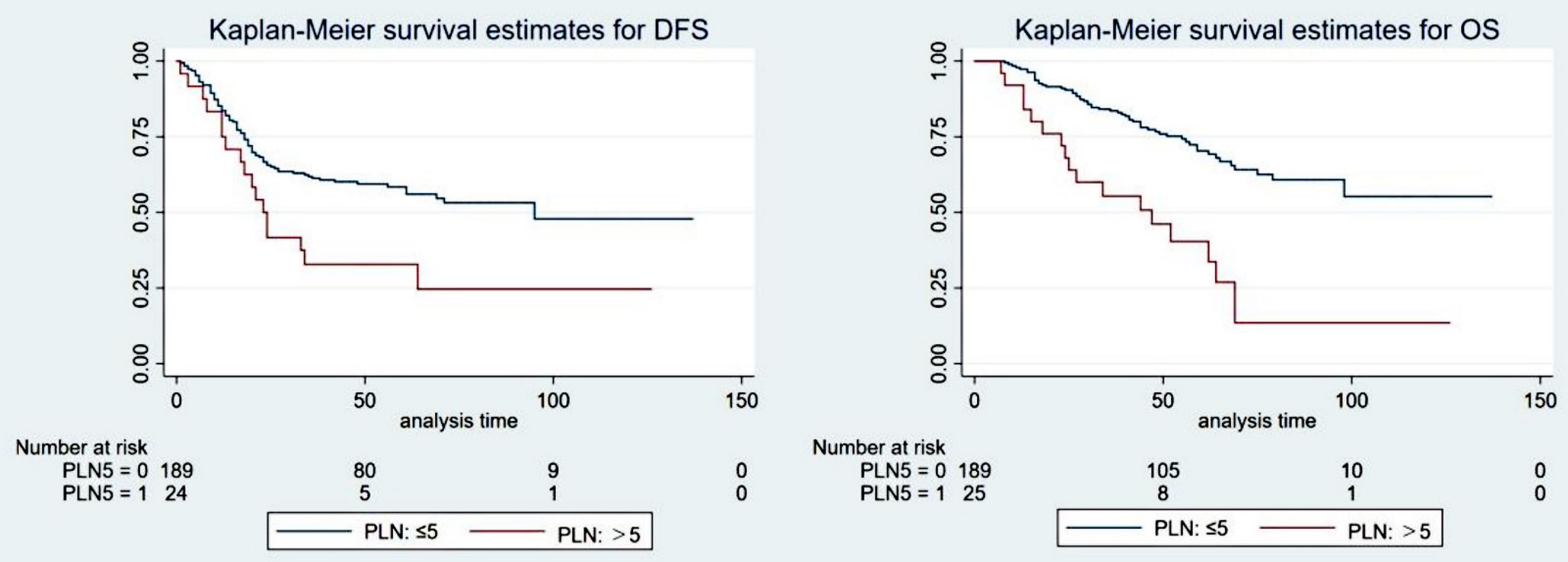
The survival curve of the ypT1-4N+ group when the optimal cutoff value of the positive lymph nodes (PLNs) was 5.

The optimal cutoff value for PLNs was confirmed to be 5 by using X-tile software. The results are detailed in the supplementary materials (**Supplementary Figure S1**). Univariate analysis showed that PLN (5 as the cutoff value) was significantly associated with DFS (5-year DFS: 58.4% vs. 31.5%, P=0.040) and OS (5-year OS: 70.3% vs. 40.4%, p<0.001). Cox multivariate analysis showed that patients with PLN ≤ 5 had significantly better DFS (HR: 2.104, 95% CI: 1.261-3.512, P=0.004) and OS (HR: 3.471, 95% CI: 1.999-6.030, p<0.001) than those with PLN>5. The patient survival curves are shown in **Figure 3**.

#### Positive Lymph Node Ratio

**Figure 4:**
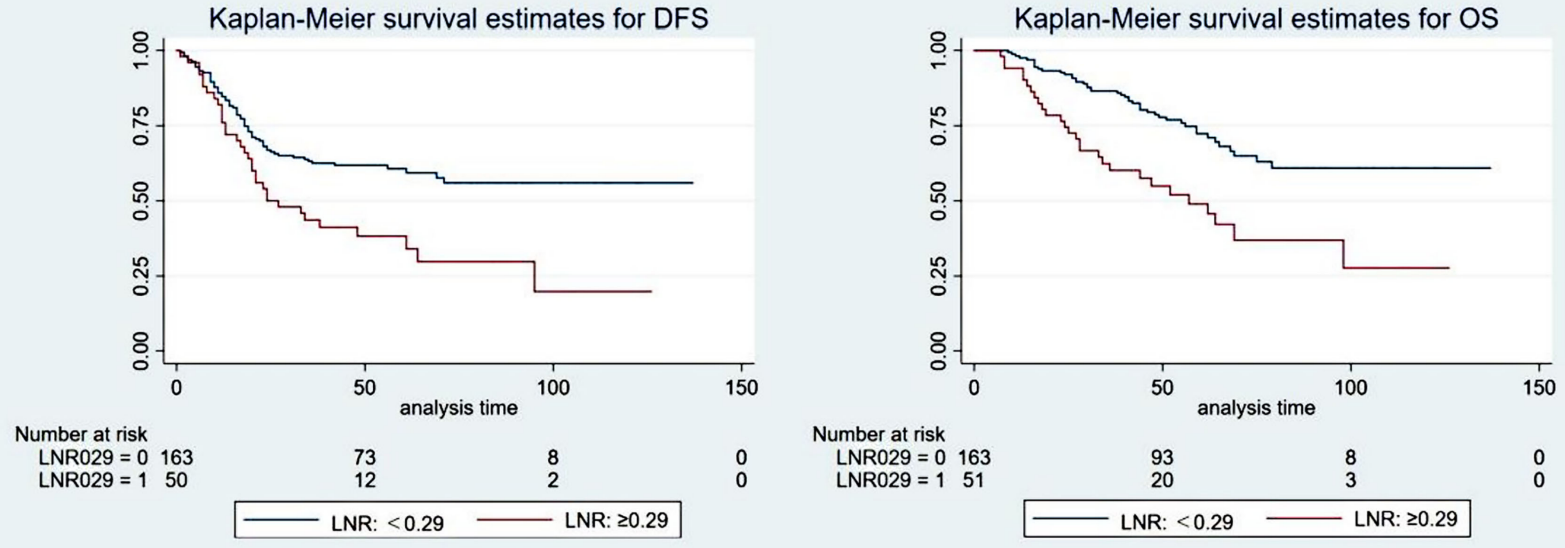
The survival curve of the ypT1-4N+ group when the optimal cutoff value of the positive lymph node ratio (LNR) was 0.29.

The optimal cutoff value for the LNR was also confirmed by X-tile software. Detailed descriptions are provided in the supplementary materials (**Supplementary Figure S2**). Univariate analysis showed that the LNR (0.29 as the cutoff value) was significantly associated with DFS (5-year DFS: 60.7% vs. 37.5%, P=0.001) and OS (5-year OS: 72.3% vs. 49.0%, p<0.001). Cox multivariate analysis showed that LNR ≥0.29 was an independent prognostic factor for poorer DFS (HR: 1.951, 95% CI: 1.289-2.955, P=0.002) and OS (HR: 2.434, 95% CI: 1.519-3.899, p<0.001). The patient survival curves are shown in **Figure 4**.

## Discussion

An accurate evaluation of lymph nodes has important reference significance for postoperative pathological staging after TME surgery and follow-up treatments, and it is a powerful prognostic factor as confirmed by several studies (12–15). The AJCC recommends that at least 12 lymph nodes should be examined for accurate tumor staging (16). However, the number of lymph nodes retrieved varies greatly under the influence of many factors (17), and approximately 30-50% of patients have fewer than 12 lymph nodes retrieved (15, 18–21). Insufficient lymph node dissection can lead to tumor understaging and poor subsequent treatment selection and ultimately result in a poor prognosis (12, 14, 15, 22–24). Unfortunately, due to controllable and uncontrollable factors, the goal of examining a sufficient number of lymph nodes is generally difficult to achieve. These factors may include the experience of the surgeon, the experience and work attitudes of the pathologist, the patient’s condition (age, sex, degree of obesity, etc.) and the characteristics of the disease (stage, tumor location and distance from the anus) (12). Previous studies have revealed that neoadjuvant therapy can lead to a significantly decreased number of lymph nodes detected (7–9). The mechanism behind this phenomenon could be radiation‐induced fibrosis, lymphocyte depletion, tissue contraction, adipocyte replacement and interstitial atrophy, making it more difficult to detect lymph nodes during surgery or pathological examination (25–27). In addition, a greater time interval to TME operations leads to an increased incidence of interstitial fibrosis, which further reduces the number of lymph nodes retrieved. The previous criteria for dissecting at least 12 lymph nodes might not be suitable for patients after nCRT.

The comparison of the different postoperative stage groups in this study showed that the total number of lymph nodes retrieved in ypT1-4N+ patients whose prognoses were poor was significantly greater than that in other groups. The reason for the increase in the total number of lymph nodes detected in this group might be as follows: the patients in this group had a poor response to nCRT, which showed poor tumor regression and the presence of positive lymph nodes, significantly reducing the difficulty of retrieving lymph nodes during surgery when compared with the other three groups. Due to the poor response of these groups to nCRT, this phenomenon mostly indicated that the patients were not sensitive to subsequent systemic treatments, their recurrence rate was high and they had worse long-term survival in spite of the increased number of lymph nodes retrieved (28, 29).

A previous study showed that TRG is significantly related to the survival of rectal cancer patients. Patients who achieved a complete remission had a significant survival benefit compared to those who did not (30). Some scholars compared the survival after neoadjuvant treatment for rectal cancer in the two groups using 12 as the cutoff point (31). The results showed that a reduction in the total number of lymph nodes retrieved did not have an adverse impact on the prognosis, suggesting that the tumor responded well to neoadjuvant therapy and had better local control. Gurawalia et al. (22) analyzed 364 rectal cancer patients diagnosed during 2010-2014, 91 of whom received neoadjuvant therapy, and the results showed that patients with fewer than 12 lymph nodes retrieved were more likely to achieve pCR (40% vs. 26%, p<0.05) and a lower TRG classification. Wang et al. (32) compared the total number of lymph nodes retrieved between the TRG0-1 group and TRG2-3 group. A subgroup analysis of the TRG0-1 group showed that the prognosis between the group with < 12 lymph nodes retrieved and the group with ≥ 12 lymph nodes retrieved was similar in terms of OS, DFS, LRFS and DMFS. However, patients with ≥ 12 lymph nodes retrieved had better OS, DMFS and DFS in the TRG2-3 group. Therefore, the author believed that retrieving sufficient lymph nodes was still necessary, especially for patients with a potentially poor tumor response. However, our study reported different outcomes. Survival analysis showed that whether the goal of 12 lymph node dissections was achieved had no difference in the long-term survival of patients regardless of the TRG group. This difference in results could be due to the differences in the treatment regimens in the study design. A total of 64.4% of the patients received interval chemotherapy in the study of Wang et al., while 0.0% did so in our study. It was noted that though the results of our study showed that the total number of lymph nodes retrieved in the TRG2-3 group was significantly more than that in the TRG0-1 group (P=0.001), the absolute difference observed was too small to be clinically significance. The same consideration was observed in the analysis of the correlation between the number of retrieved nodes and downstaging after nCRT. Large randomized control trials are necessary to test these hypotheses.

At present, the influence of the total number of lymph nodes retrieved after neoadjuvant treatment on the prognosis of patients with rectal cancer is still controversial. Several studies found that dissection of < 12 lymph nodes did not adversely affect survival (7, 33, 34). In contrast, Narayanan et al. (35) extracted data from patients diagnosed with rectal cancer during 2004-2014 from the NCDB. They found that retrieving ≥ 12 lymph nodes in patients who did not reach ypCR after neoadjuvant therapy can improve OS but not in patients who achieved ypCR. In our study, there was no statistically significant difference in DFS and OS between the group with < 12 lymph nodes and the group with ≥ 12 lymph nodes. Subsequent Cox multivariate analysis showed that 7 may be the best cutoff value for the total number of lymph nodes retrieved after nCRT. Other more sensitive lymph node-related parameters should be further evaluated, such as PLN and LNR. PLN and LNR have been considered important prognostic factors for RC (36–39).

Several studies have shown that the number of positive lymph nodes is significantly reduced or even disappears after neoadjuvant treatment, achieving the goal of downgrading and indicating a better prognosis. In a meta-analysis including 7 studies, the number of positive lymph nodes decreased significantly after neoadjuvant therapy (40). Another meta-analysis indicated that nCRT could reduce the number of positive lymph nodes by an average of 0.7 (14). The results of our study showed that when 5 was taken as the best cutoff value for PLN, the difference in survival between the two groups was statistically significant.

The current 8th edition of the NCCN guidelines does not formulate new postoperative staging guidelines for patients who underwent neoadjuvant therapy. The guidelines use 4 as the cutoff value for N1 and N2 staging, while the results of our study show that 5 was the best cutoff value for N staging in patients who received neoadjuvant therapy. The cutoff value indicated that neoadjuvant therapy might have the potential to posteriorly shift the cutoff value of N staging. If neoadjuvant therapy can be incorporated into the AJCC staging system, it will be more conducive to accurately assessing the prognosis of patients and guiding follow-up treatments.

In various recent studies, the LNR has been proven to be an independent prognostic indicator, and a lower LNR indicates a better survival rate (41–43). In a study of 605 patients with rectal cancer, Dekker et al. (44) found that LNR ≥ 0.6 was significantly related to OS and LRFS, while N stage was not. Other studies have shown that the LNR has prognostic value in patients with ypN+ (stage III) rectal cancer regardless of the total number of lymph nodes retrieved (12, 45). In our study, there were significant differences in survival when 0.29 was used as the best cutoff value for the LNR. If it is difficult to retrieve the recommended number of lymph nodes from patients due to difficulty in lymph node dissection during surgery, the LNR can be used as a prognostic factor for these patients.

This study is a retrospective analysis. Although the sample size was large, selection bias was still unavoidable. Pathological factors have a great influence on the detection of lymph nodes, but the analysis of pathological techniques and other factors was absent in this study. The time span was 9 years in this study, and the prognosis was affected by the development of new radiotherapy techniques, chemotherapy regimens and other treatment methods. However, the above factors were included in the multivariate analysis, which offset the deviation caused by the development of treatments to a certain extent.

## Conclusion

For LARC patients undergoing nCRT, the recommendation of dissection of ≥ 12 lymph nodes may not be appropriate. The recommends that at least 7 harvested lymph nodes may be more appropriate. New lymph node-related parameters, such as PLN and LNR, provide major new insights into predicting the outcomes of LARC patients undergoing nCRT. 5 positive lymph nodes represents a stronger independent prognostic indicator and can be treated as the best cutoff value for N1 and N2 staging when neoadjuvant therapy can be incorporated into the AJCC staging system. In the future, further prospective controlled studies are required to verify this finding.

## Data Availability Statement

The original contributions presented in the study are included in the article/**Supplementary Material**. Further inquiries can be directed to the corresponding authors.

## Ethics Statement

The studies involving human participants were reviewed and approved by the Fujian Medical University Union Hospital Ethic Review Board (reference number: 2021KY171). The requirement for consent was waived by the ethics committee due to the retrospective nature of the study.

## Author Contributions

Conception and design: ZL, AL, and BX. Administrative support: JS, AL, and BX. Provision of study materials or patients: XL, RZ, and CC. Collection and assembly of data: ZL, JS, and AL. Data analysis and interpretation: ZL, JS, and AL. Writing – original draft: ZL. Writing – review and editing: AL. All authors contributed to the article and approved the submitted version.

## Funding

Benhua Xu, Joint Funds for the innovation of science and Technology, Fujian province (2017Y9061) and Fujian provincial health technology project (2021CXA011).

## Conflict of Interest

The authors declare that the research was conducted in the absence of any commercial or financial relationships that could be construed as a potential conflict of interest.

## Publisher’s Note

All claims expressed in this article are solely those of the authors and do not necessarily represent those of their affiliated organizations, or those of the publisher, the editors and the reviewers. Any product that may be evaluated in this article, or claim that may be made by its manufacturer, is not guaranteed or endorsed by the publisher.

## Supplementary Material

Supplementary Figure 1 The optimal cutoff value of positive lymph nodes (PLNs) by X-tile software. The optimal cutoff value is shown on a histogram (left panel) and a summarized results table (right panel). of the entire cohort. The blue and gray bars/characters designate low-risk and high-risk subsets, respectively. The optimal cutoff value of PLNs is 5 based on the lowest p values (0.0005) and the maximum χ2 (19.0055) of log-rank tests.

Supplementary Figure 2 The optimal cutoff value of the positive lymph node ratio (LNR) by X-tile software. The optimal cutoff value is shown on a histogram (left panel) and a summarized results table (right panel). The blue and gray bars/characters designate low-risk and high-risk subsets, respectively. The optimal cutoff value of LNR is 0.29 based on the lowest p values (0.0047) and the maximum χ2 (14.5450) of log-rank tests.
