# Supplementary figures and images for: The Effect of Lymph Node Harvest on Prognosis in Locally Advanced Middle-Low Rectal Cancer After Neoadjuvant Chemoradiotherapy
Source: Front Oncol. 2022 Feb 15;12:816485. doi: 10.3389/fonc.2022.816485 (PMC8886163; doi:10.3389/fonc.2022.816485)

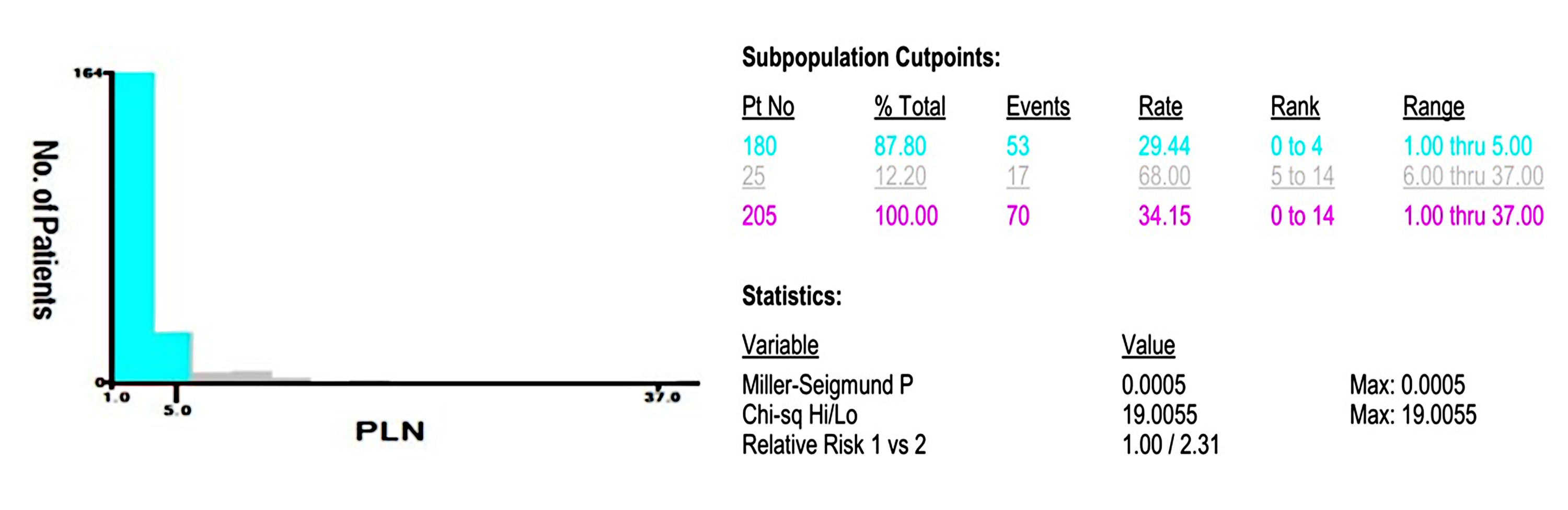

Supplement: Supplementary Figure 1 — The optimal cutoff value of positive lymph nodes (PLNs) by X-tile software. The optimal cutoff value is shown on a histogram (left panel) and a summarized results table (right panel). of the entire cohort. The blue and gray bars/characters designate low-risk and high-risk subsets, respectively. The optimal cutoff value of PLNs is 5 based on the lowest p values (0.0005) and the maximum χ2 (19.0055) of log-rank tests. [file Image_1.tif]

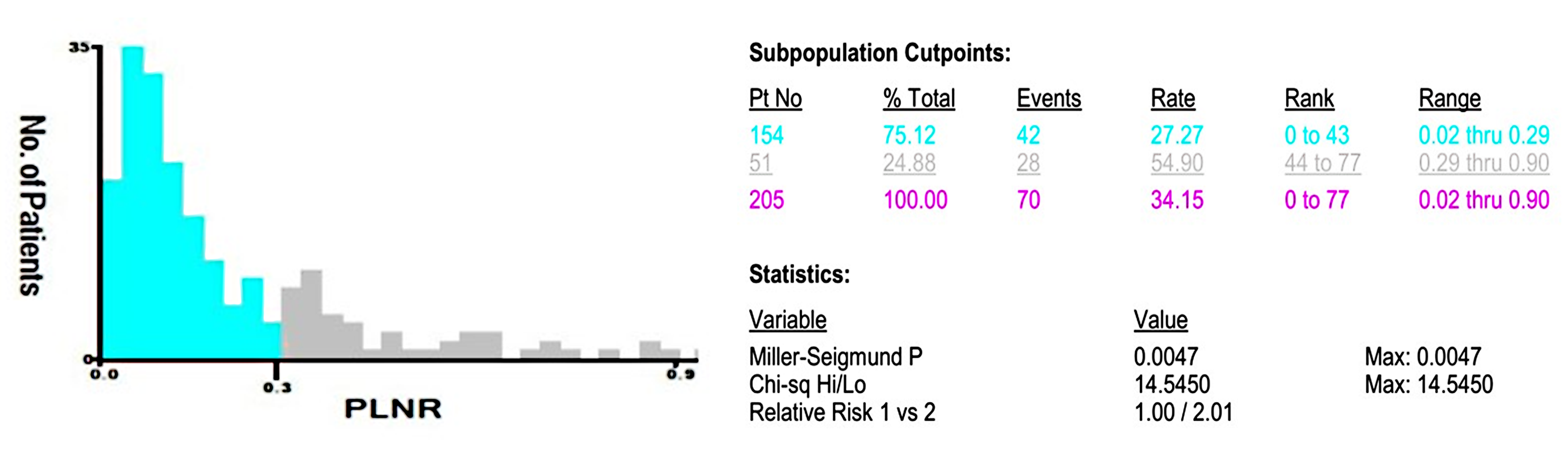

Supplement: Supplementary Figure 2 — The optimal cutoff value of the positive lymph node ratio (LNR) by X-tile software. The optimal cutoff value is shown on a histogram (left panel) and a summarized results table (right panel). The blue and gray bars/characters designate low-risk and high-risk subsets, respectively. The optimal cutoff value of LNR is 0.29 based on the lowest p values (0.0047) and the maximum χ2 (14.5450) of log-rank tests. [file Image_2.tif]
